# Adversity, Trauma Symptoms and the Effectiveness of an Australian Individualised Developmental Trauma Intervention Program

**DOI:** 10.1007/s40653-024-00674-x

**Published:** 2025-01-16

**Authors:** Pamela Smith, Sonia Sharmin, Dallas Ambry, Allison Cox, Erin Hambrick, Margarita Frederico, Holly Mosse

**Affiliations:** 1 Berry Street Take Two Victoria, Richmond, Australia; 2 Department of Occupational Therapy and Social Work and Social Policy, School of Allied Health, Human Services and Sport, College of Science, Health and Engineering, La Trobe University, Bundoora, Victoria Australia; 3 University of Missouri, Kansas City, Kansas City, MO USA

**Keywords:** Developmental trauma, Adversity, Interventions, Maltreatment, TSCC, TSCYC

## Abstract

This study evaluated the effectiveness of a therapeutic intervention program Take Two; designed to address developmental trauma experienced by Child Protection clients in Victoria, Australia. Replicating a 2010 evaluation study of the program, we utilised a Time 1—Time 2 design to identify the impact of tailored Take Two treatments informed by the Neurosequential Model of Therapeutics’ (NMT™). Change in the overall sample was measured by the Trauma Symptom Checklist for Children (TSCC; ages 8–16 years) and Trauma Symptom Checklist for Young Children (TSCYC; ages 3—12 years). In addition, a sub-cohort of children with severe adverse infant experiences was identified using a developmental history of adversity tool; Part A of the Neurosequential Network’s Neurosequential Model of Therapeutics (NMT™) Metric. Treatment effects were also evaluated to determine the extent to which this potentially more vulnerable subgroup was improving. Significant improvement was found in the TSCC cohort (8–16 years) with effect sizes ranging from small to medium (*d* = 0.23–0.54) on TSCC sub-scales. The largest effects were found on Anxiety (0.54), which moved from sub-clinical to non-clinical. The TSCYC cohort (3–12 years) showed significant symptom reduction on all trauma scales with medium sized effects (*d* = 0.44–0.53), and the largest effect on Posttraumatic Stress-Total (0.53). In the sub-cohort experiencing moderate-to-severe adversity in infancy, effect sizes were small to medium (*d* = 0.15–0.59). Take Two interventions were associated with significantly reduced trauma symptoms even when children’s adverse experiences in infancy were moderate to severe, highlighting the benefits of NMT™ guided systemic and individually tailored therapeutic interventions.

In Australia, 174,000 children (1 in 32) were in the Child Protection system between 2021–2022, including children with cases under investigation, 45,500 cases of substantiated maltreatment, children on care and protection orders, or children in out-of-home care (OOHC) (Australian Institute of Health & Welfare, 2023). This group includes an overrepresentation of infants, children from remote areas, and Aboriginal or Torres Strait Islander children (Australian Institute of Health & Welfare, 2020). Children and young people in the Australian OOHC system often have histories of extreme and multiple forms of abuse, abandonment, and/or neglect (Harris et al., 2024), alongside other forms of adverse childhood experiences (ACEs) (Felitti et al., 1998). Trauma and attachment adversity is associated with the development of psychological difficulties such as depression, anxiety, anger, posttraumatic stress symptoms, attentional, behavioural, emotional and relational dysregulation, functional impairments, maladaptive behaviour, negative long-term social and emotional outcomes and early mortality (Grummitt et al., 2021; Kim & Cicchetti, 2010; Kisiel et al., 2014; Spinazzola et al., 2018). The purpose of this study is to contribute to research on interventions for developmental trauma exposure by examining the effectiveness of a therapeutic program called Take Two at reducing trauma-related symptoms in a sample of children aged three to 16 years, including children with documented histories of severe early-life child abuse and neglect.

## Maltreatment, Adversity, and Developmental Trauma

It has long been established that ACEs, which include maltreatment (hereafter adversity) have detrimental effects on children’s long term physical and psychological health (Felitti et al., 1998; Hambrick et al., 2019a). Adversity is multidimensional (Cicchetti & Toth, 1995): type, frequency, chronicity, co-occurrence, age, and severity all influence outcomes. Type, multi-type and severity of ACEs have been associated with distinct psychological symptoms (English et al., 2005) and neurological changes (Teicher & Samson, 2016). Type, severity and chronicity of adversity may be the most predictive (English et al., 2005; Warmingham et al., 2019) and yet timing of adversity also significantly influences developmental pathology, with perinatal adversity and/or relational poverty linked to more disruptive developmental trajectories than adversity occurring later in life (Hambrick et al., 2019b; Russotti et al., 2021). Traumas experienced during critical early developmental stages can negatively affect the brain’s stress response system, influencing the organisation of children’s neurodevelopment and therefore leading to poor functioning later in life (Perry, 2008). Despite documented effects of early and severe adversity, relationally rich experiences such as attuned caregiving and social support can provide a buffer to the impact of adversity (Hambrick et al., 2019b), making early systemic interventions vital for Child Protection clients (Cox et al., 2021a).

Developmental trauma refers to many different types of adverse experiences which may occur from conception onwards in the context of caregiving relationships, to the potential detriment of the child’s neurobiological development (van Der Kolk, 2005). The interpersonal nature of these traumas – and the typical lack of relationally positive supports present for children when the traumas are experienced – can result in a host of outcomes that may persist into adulthood. Wide variations in individual symptom profiles occur because the nature, timing, chronicity, and severity of early life trauma experiences vary amongst children (DePierro et al., 2022), thus requiring individually tailored treatments (Ford, 2021; Tarren-Sweeney, 2013). We henceforth use the term developmental trauma in this paper to describe not only the experience but the potential impact of experiencing multiple types of trauma (Denton et al., 2017; Ford, 2023; Tarren-Sweeney, 2013).

## Trauma Treatment and Evaluation

A review of the literature found two effectiveness studies measuring trauma outcomes in similar populations and community treatment settings to Take Two clients. Dauber et al. (2015) evaluated an agency-based trauma recovery program for children and adolescents (8–17 years) with complex trauma (*n* = 31), using multiple therapies in a phase-based approach (Dauber et al., 2015). These therapies included attachment based, cognitive-behavioural and creative arts approaches, with additional training given to clinicians in Trauma-Focused Cognitive Behavioural Therapy (TF-CBT) (Cohen, 2006) and Attachment, Self-Regulation and Competency (ARC) (Kinniburgh et al., 2005). Utilising the Trauma Symptom Checklist for Children (TSCC, Briere, 1996) outcome measure, this program demonstrated significant improvement in trauma symptoms (Dauber et al., 2015). The Massachusetts Child Trauma Project (*n* = 842) employed TF-CBT, ARC and Child-Parent Psychotherapy (CPP) (Lieberman, 2008) for children (0–18 years) in the Child Protection system with significant reductions in PTSD symptoms (Bartlett et al., 2018). These two studies provide information about age, care settings, trauma types and intervention outcomes for children who experience developmental trauma contributing to incremental improvement in knowledge about what can work in this population, however there is much to be discovered about effective treatment in real-world settings, where relatively little is known.

Evidence-based trauma treatments prove efficacious in controlled trials, but few studies are conducted in community settings (Jensen et al., 2014) where they are less likely to demonstrate efficacy (Weisz et al., 2013). In addition, children with developmental trauma presentations, developmental disorders and intellectual disabilities, infants, and children from different cultures are often excluded, missing or have high dropout rates from RCTs (Chorpita, 2003; Frederico et al., 2019), as seen in Leenarts et al (2013) systematic review of evidence based interventions and a recent TF-CBT study (Ross et al., 2021), suggesting the findings may not be generalisable to community settings. To take the issue a step further, systematic reviews suggest there is insufficient evidence to rely on single, discrete interventions for Child Protection clients (Fraser et al., 2013) and/or developmental trauma (Ford, 2021). Organisational theories of development indicate that interventions are likely to fail when not matched to the child’s unique developmental and cultural needs (Hambrick et al., 2016). More research in community-based therapeutic settings is required to help determine evidence-based practice for this population.

Perry (2009) and Ford recommend matching interventions to children’s individual therapeutic needs in a ‘precision health approach’ (Ford, 2021, p. 1) for developmental trauma, which the NMT™ enables via thorough neurodevelopmental assessments. The NMT™ is a developmentally sensitive, neurobiologically informed framework for clinical intervention planning and delivery (Perry, 2006), designed to complement not replace other metrics, assessment components, and evidence-supported interventions (Perry & Dobson, 2013)*.* Emerging effectiveness research for NMT™ guided interventions is reported in eight studies (Brank et al., 2020; Cox et al., 2021b; Hambrick et al., 2018), including a recently published randomized controlled trial (White et al., 2023).

Neurodevelopmentally informed trauma and attachment therapies are recommended for developmental trauma, which in accordance with phase-based approaches may begin with relationship building, somatic, emotion and behaviour regulation skills and systemic interventions to establish safety and stabilization. Ensuring children have stable caregivers to provide the relational attunement necessary to assist with attachment-based and self-regulation interventions is foundational (Frederico et al., 2019). Once safety and regulation skills are established, therapeutic interventions can progress to trauma processing and integration phases (Ford, 2021).

These recommendations, while grounded in research, can pose challenges for program implementation given the complexity of such endeavours. Moreover, evaluating intervention effectiveness when children’s histories of adversity and clinical presentations are heterogeneous is challenging, resulting in major gaps in our understanding of how to best intervene with Child Protection clients.

## The Take Two Program

Berry Street is a large not-for-profit community service organisation in Victoria, Australia and amongst other programs delivers the Take Two program funded by the Department of Families Fairness and Housing. Take Two is a statewide innovative therapeutic and research program addressing the impact of severe abuse, neglect, or adverse experiences on statutory Child Protection clients 0–17 years, who are often living in out-of-home care settings such as foster care, kinship care and residential care homes. The Intensive Therapeutic Service (ITS) is Take Two’s primary service arm for children referred from Child Protection with substantiated experiences of abuse and/or neglect. Take Two operates from a trauma, attachment and developmentally informed theoretical base applying an ecological and cultural perspective, and works to overcome the barriers Child Protection clients face accessing therapeutic services (Frederico et al., 2019). Take Two is overseen by a consortia led by Berry Street in partnership with Mindful, La Trobe University, and the Victorian Aboriginal Child Care Agency. Before the program’s establishment in 2004, there was no statewide therapeutic service in Victoria for Child Protection clients (Jackson et al., 2009).

By the end of 2011, Take Two was Site-Certified in use of the Neurosequential Model of Therapeutics (NMT™, Perry, 2006). NMT™ clinical intervention planning is facilitated by a set of clinical practice tools (i.e., NMT Metrics) to assess children’s developmental experiences and how these experiences may have influenced their current functioning. The NMT™ prioritizes building safe and secure relationships at all levels of a child’s social ecology (e.g., caregivers, teachers, mentors, coaches, friends, faith and cultural community), while also sequencing the delivery of trauma-informed and other developmental interventions based on a child’s current functioning in four broad domains: sensory, self-regulatory, relational, and cognitive.

Take Two employs a suite of evidence-based and other practice-informed interventions, the timing and intensity of which are informed by NMT intervention planning. Individualised treatment plans are developed using the Neurosequential Network’s Neurosequential Model of Therapeutics Metric Tool (NMT™ Metric Tool; The Neurosequential Network, 2023), and the timing of therapeutic techniques are matched to the developmental needs of the child (Perry, 2008). Interventions begin with ensuring those in the child's network are supported to be therapeutic agents of change, beginning with the therapeutic web (Perry & Dobson, 2013). This is predicated on recognising that to heal and recover, children require safe environments, and adults with responsibility for ensuring this occurs may need psychoeducation and support. Take Two interventions at a systems and community level include psychoeducation with case managers, schools, and other professionals to facilitate and maintain a supportive social environment around the child. Enlisting the child's community of culture is equally pivotal in supporting a relational environment of recovery. Culturally informed assessments and interventions for Aboriginal and Torres Strait Islander children such as the Cultural Connection Assessment Tool designed through Take Two (Aldobasic et al., 2021) are embedded into the program. Interventions with the child, family, kinship, and other carers that are commonly applied within Take Two include EMDR (Shapiro, 2001), CPP (Lieberman, 2008), play therapy, child-focused parent therapy, family therapy, somatosensory activities and creative arts therapies (Frederico et al., 2019).

At Take Two, we complement the use of the NMT™ Metric Tool with a suite of clinical measures including the Trauma Symptom Checklist for Children (TSCC, Briere, 1996) and the Trauma Symptom Checklist for Young Children (TSCYC, Briere, 2005), seeking to capture relevant developmental trauma sequelae at the start of treatment, inform treatment planning and measure intervention progress. The TSCC and TSCYC were utilised in a discrete study from 2004–2007 (*n* = 74) as part of a program evaluation with promising results (Frederico et al., 2010), providing the impetus for this study 15 years later.

## Current Study

This study fills two important gaps in the literature. First, it contributes to the overall literature regarding the effectiveness of holistic, sequenced interventions for a diverse and high need group of children with developmental trauma who are Child Protection clients. Second, it examines whether positive effects are evidenced even when children’s adverse experiences during infancy, a potential critical period for their overall development, are severe. Moreover, we will test the hypothesis that Take Two ITS interventions reduce trauma symptoms in Child Protection clients from intake to post-treatment, even when their adverse experiences in infancy have been severe.

## Method

### Procedures

This study utilised a Time 1- Time 2 design to study client improvement July 2007- July 2019. At intake, children’s developmental functioning and mental health symptoms are assessed by their clinician using a variety of clinical tools and assessments. This is repeated at review and closure. Resulting data is stored in a repository, from which this study’s data was accessed.

### Inclusion Criteria

The sample included Take Two clients from ITS with confirmed experiences of adversity referred by Child Protection services. An average Take Two ITS intervention is 14 months, with clinical measures administered every 6 months. Clients who had two or more valid measurements on measures of trauma-related functioning completed between a five and 16-month period of intervention were included.

### Exclusion Criteria

Clients whose clinical measures fell outside of the 5–16 month intervention period were excluded. Incomplete administration or measures with missing data or incorrect dates were excluded, including measures of trauma-related functioning that were completed by raters other than the primary carer (such as teachers or case workers). Where more than one carer had completed the TSCYC measure, measures from the same rater at (T1) and (T2) were prioritised. In 89.59% of cases raters were the same at T1 and T2. In 10.41% of cases raters changed between administrations: this data was included. Where children (8–12 years) had both self-report and carer reports available, the TSCC self-report was utilised.

### Sample

Approximately 1550 children commenced an ITS intervention at Take Two from 2007–2019, completing a minimum of five months engagement with the service; among them 44.5% were female, 55.5% male and 0.1% other gender, 24.8% were of Aboriginal or Torres Strait Islander origin and the average age was 8.6 years (M = 8.6, SD = 4.2). 44.5% of them were living in home-based care, 5.5% were in residential care, while 41.6% had no known placement at the time of assessment.

Application of the exclusion and inclusion criteria resulted in a final sample of 235 children with data available for analysis and whose demographic data is shown in Table 1. A lack of measures completed in the intervention period were mainly responsible for the reduced sample. In such a complex population, factors such as engagement, attrition and placement instability make obtaining timely assessments challenging, also contributing to the reduced sample. Children’s data were split into sub-groups based on age and depending upon the availability of measures (TSCC or TSCYC) that fit the inclusion criteria. Data from *n* = 91 clients applying the TSCC aged 8–16 years with a mean age of 11.5 years and *n* = 144 clients applying the TSCYC aged 3–12 years with a mean age of 6.8 years were separately analysed to evaluate improvement in their trauma-related symptoms. A sample of *n* = 15 clients aged 8–16 years and *n* = 102 children aged 3–12 years were available for inclusion in analyses using the NMT™ metric evaluating the effects of moderate to severe adversity on outcomes.

**Table 1:** Demographics and developmental adversity of the sample at T1 (*n* = 235)

Variable	*TSCC*		*TSCYC*	
	*n* = 91	%	*n* = 144	%
Gender^a^				
Female	46	50.5	52	36.1
Male	45	49.5	92	63.9
Aboriginal or Torres Strait Islander				
Yes	13	14.3	30	20.9
No	78	85.8	114	79.1
Age in years (M/SD)	11.5 (1.74)		6.8 (2.41)	
Location				
Rural	32	35.2	72	50.0
Metropolitan	59	64.8	72	50.0
Care environment				
Kinship care	29	31.9	60	41.7
Home based care^b^	22	24.2	23	16.1
Living with one or both parents	6	6.6	4	2.8
Permanent care	1	1.1	-	-
Family group home^c^	1	1.1	-	-
NMT metric Part A: developmental adversity				
During infancy^d^				
None/Minimal	5	5.5	8	5.6
Mild	17	18.7	34	23.6
Moderate	12	13.2	93	64.6
Severe	3	3.3	9	6.3

^a^ Other gender category isn’t included here due to *n* = 0 cases

^b^ Home Based Care in this study includes children in foster care, general, intensive, and specialised home-based care, and adolescent community placement

^c^ Family Group Home differs to Residential Care in this study

^d^ 6–23: None/Minimal; 24–41: Mild; 42–59: Moderate; 60–72: Severe

### Demographic Information

**Fig. 1:**
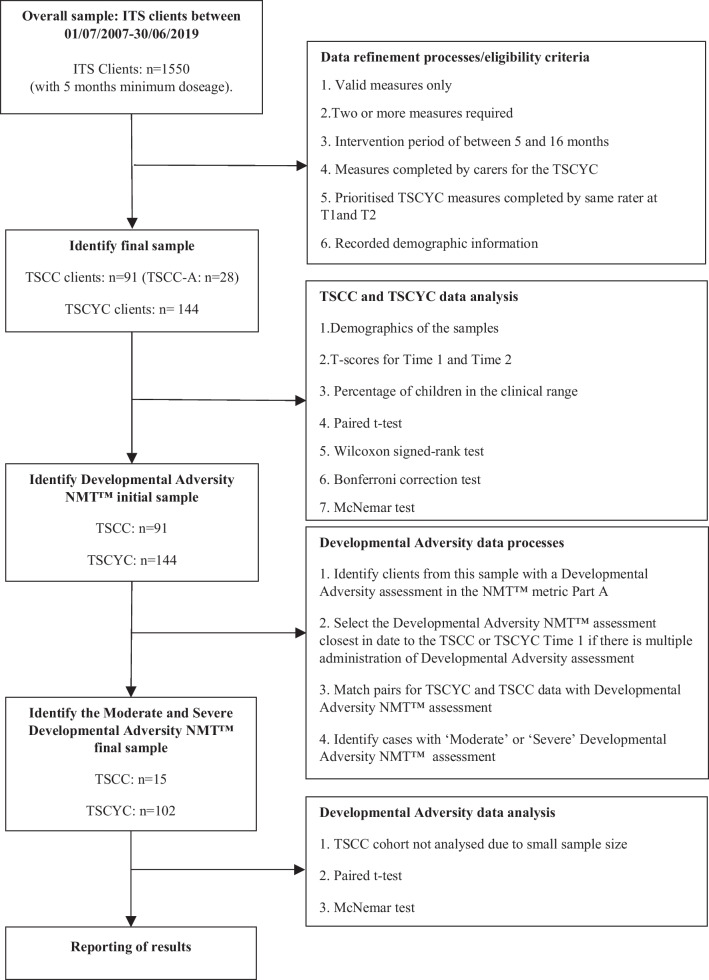
Participant flow through the study from selection to analysis

At Time 1, client age, gender, Aboriginal and Torres Strait Islander status, type of measure (TSCYC, TSCC, NMT Part A), type of placement (e.g., foster, kinship) and whether the client was treated at rural or metropolitan team was recorded. Disability data was not available (Fig. 1).

## Measures

### Trauma-Related Symptoms: the Trauma Symptom Checklists for Children and Young Children (Briere, 1996, 2005)

Given that manifestations of trauma exposure change as children age and that young children are not reliable reporters of their mental health symptoms, we used two different versions of our primary clinical measure: the Trauma Symptom Checklist for Children (TSCC; child self-report ages 8–16 years, Briere, 1996) and the Trauma Symptom Checklist for Young Children (TSCYC; carer report ages 3–12 years, Briere, 2005). Therefore, results are presented separately for children whose trauma symptoms were described by their carers using the TSCYC (3–12 years) than children who self-reported trauma symptoms using the TSCC (8–12 years).

Although the complex aetiology and presentation of developmental trauma challenge the scope of a single measure to capture the full range of symptoms and developmental difficulties experienced (Denton et al., 2017), the TSCC and TSCYC are efficient, standardised measures of a range of trauma symptomatology (Wolpaw et al., 2005). The TSCC measures trauma symptoms on six scales: Anger (ANG), Anxiety (ANX), Depression (DEP), Posttraumatic Stress (PTS), Dissociation (DIS) and Sexual Concerns (SC). The PTS scale measures posttraumatic symptoms such as intrusive, avoidance and hyperarousal symptoms but is not considered a comprehensive measure of posttraumatic stress disorder (PTSD). *T*-scores at 65 or above are clinically significant indicating that these symptoms are likely to negatively impact the child, while scores between 50 and 64 are considered sub-threshold elevations. SC scores at or above 70 are considered clinically significant, below 70 is non-clinical (no sub-clinical category exists for SC). The TSCC-Alternate version (Briere, 1996) excludes items that constitute the SC scale, accommodating children and Aboriginal clients for whom these questions are distressing or culturally inappropriate. TSCC has an inbuilt validity scale, excluding responses that over or under-report symptoms.

The TSCYC assesses trauma symptoms across six scales: PTS, SC, ANX, DEP, DIS and Anger/Aggression (ANG/AGG). *T*-scores at 70 or above are clinically significant, scores between 65 and 69 are sub-threshold. Two validity scales (Response Level and Atypical Response) are included in the measure.

The TSCC was validated in a sample of 3008 children aged 8–12 years with good internal consistency on Cronbach’s alpha ranging from 0.77—0.89 on the clinical scales and 0.84 for the total scale (Briere, 1996). Clinical samples have also demonstrated reliability and validity (Briere, 1996; Lanktree et al., 2008). Reliability and validity of the TSCYC has been established in several studies (Briere et al., 2001; Milot et al., 2010) with high internal consistency (Briere, 2005). Moderate convergent and discriminant validity between the TSCC and TSCYC indicates different perspectives on the child’s symptomatology (Lanktree et al., 2008).

### History of Adversity: NMT™ Metric Tool (the Neurosequential Network, 2023)

The NMT™ Metric Tool prompts clinicians to gather and rate information about a child’s developmental history and current functioning to assist with intervention planning and monitoring. The NMT™ Metric Tool consists of four parts: Part A: Developmental Adversity, which prompts clinicians to collect information regarding adversities the child experienced during discrete developmental windows, Part B: Developmental Relational Health, which prompts clinicians to collect information regarding the quality of relational experiences the child had during the same developmental windows as Part A; Part C: Current Functioning, which prompts clinicians to rate children’s current developmental capacities across a range of brain-mediated functions (sensory, self-regulatory, relational and cognitive), and Part D: Current Relational Health, which prompts clinicians to report on a child’s current relationally positive experience across a variety of contexts, including home, culture, and school. Evidence for good reliability (internal consistency), and validity (predictive) of the NMT™ is reported by Hambrick et al. (2019) in four studies. Additionally, Part A scores have been found to correlate with children’s functioning in predictable ways, with higher Part A scores (indicative of more adversity) predicting poorer outcomes for children (Hambrick et al., 2019b).

Clinicians are provided with extensive training in Metric Tool use throughout the NMT™ certification process (Phase I certification training is approximately 150 h). The Neurosequential Network conduct at least one Fidelity Exercise per year, where all Metric Tool users across different countries are given the same anonymised case (client) data with which to complete the metrics. Clinician performance in this exercise yields a fidelity rating of None, Low, Acceptable, or High. This rating reflects the degree of interrater reliability between the clinician and expert NMT™ Metric Tool raters. Clinicians whose Metrics were included in this study were NMT Phase I Certified and had achieved a an “acceptable” or “high” fidelity rating or were in an advanced stages of completing the certification process.

Only data from Part A, Developmental Adversity, was used in this study. Six types of potentially adverse experiences are recorded for several developmental periods of a child’s life in Part A: quality of primary caregiving, caregiver drug/alcohol use, neglect, domestic violence, transitions/chaos, and other traumas. The severity of these experiences is rated in the following manner: None/Minimal (1–3), Mild (4–6), Moderate (7–9), to Severe (10 − 12). When there is a lack of reliable developmental history to inform a rating, clinicians are advised to rate the experience as Neutral (6–7). Scores rated in the severe category reflect experiences of profound trauma and adversity. The developmental periods are: Intrauterine (conception to birth), Perinatal (birth to ≥ 2 months), Infancy (3 to ≥ 12 months), Early Childhood (13 months to ≥ 3 years), Childhood (4 to ≥ 10 years) and Youth (11 to ≥ 18 years) (Hambrick et al., 2019b). The developmental period chosen as the focus for this study was what occurred in the Infancy period, given that early life trauma as measured by the Metric Tool has been found to have a consistently meaningful correlation with later life functioning (Hambrick et al., 2019a). As this study begins in 2007 and NMT™ was introduced at Take Two in 2010, not all clients in this sample had NMT data. Children without Part A NMT™ Metric Tool data were excluded in this part of the analyses that examined how children with moderate to severe adversity improved over the course of Take Two ITS intervention (see Table 1).

### Data Analysis

All analyses were conducted using IBM Statistical Package for the Social Sciences (SPSS) Statistics version 27. Descriptive statistics were used to present the demographic characteristics and developmental adversity of this sample at T1. The distribution of data for normality was assessed to ascertain the use of parametric and non-parametric tests. To test for changes in reported symptoms from T1 to T2, paired sample *t*-tests and Wilcoxon signed-rank tests were utilised on all TSCC and TSCYC scales. The Bonferroni corrected *p*-value (statistical adjustment for multiple comparisons) was applied to reduce the risk of type 1 errors. Cohen’s *d* (Cohen, 1992) was calculated and used r = z/√n (for the nonparametric test) as a measure of effect size for each TSCC and TSCYC subscales (Tomczak & Tomczak, 2014). Cohens’ *d* effect size estimates are small (*d* = 0.0–0.20), medium (*d* = 0.30–0.50), and large (*d* = 0.60–0.80). Effect size estimate measured by r = z/√n can be interpreted as small (0.1), moderate (0.3), and large (0.5 and above). The McNemar test is used to compare paired nominal data (such as T1 to T2) and in this study was conducted to compare the proportion of the sample falling above the clinical range in the TSCC and TSCYC symptom scales from T1 to T2. These tests were then applied to the sub-cohorts who had experienced moderate or severe developmental adversity during infancy (identified by NMT™ Metric Tool Part A). The resulting developmental adversity TSCC sample was too small (*n* = 15) to include in further testing, limiting our developmental adversity analysis to the TSCYC sample.

### Ethics

Ethics approval for the data captured for this study was received from La Trobe University, Victoria, Australia HEC 21070.

## Results

### Demographics

Demographic characteristics of the sample are depicted in Table 1. Of note was the larger proportion of children in the younger TSCYC cohort identified as male (63.9%), Aboriginal or Torres Strait Islander (20.9%) and living in kinship care (41.7%). The TSCYC cohort were also reported as experiencing more severe adversity during infancy than the TSCC cohort. This sample contains more males (49.5% TSCC and 63.9% TSCYC) than females compared to the larger sample, no children identifying as other gender and slightly fewer Aboriginal or Torres Strait Islander children than the larger sample (14.3% TSCC and 20.9% TSCYC). Most children were living in kinship care (31.9% TSCC and 41.7%TSCYC).

### TSCC Cohort

**Fig. 2:**
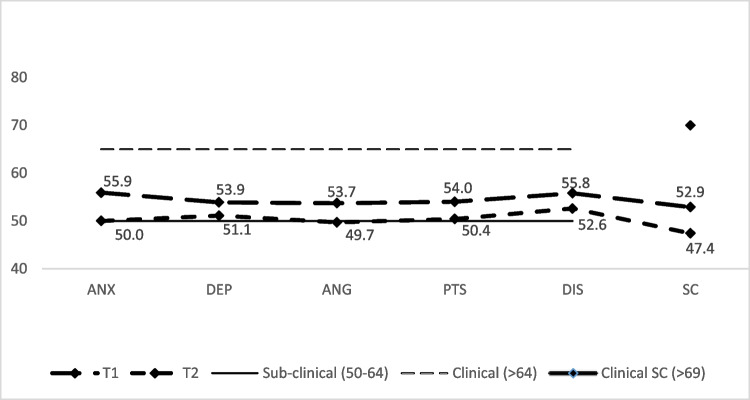
TSCC Clinical change in symptoms from T1 to T2 in Take Two clients

In Table 2, the paired sample t-test and Wilcoxon signed-rank test show significant improvement in trauma symptoms across every clinical scale from T1 to T2; namely anxiety (ANX) (*t* (91) = 5.13, *p* < 0.001, *d* = 0.54), depression (DEP) (*t* (91) = 2.18, *p* = 0.032, *d* = 0.23, anger (ANG) (z (91) = 3.84, *p* < 0.001, *r* = 0.38), posttraumatic stress (PTS) (*t* (91) = 3.38, *p* = 0.001, *d* = 0.35), dissociation (DIS) (*t* (91) = 3.08, *p* = 0.003, *d* = 0.32) and sexual concerns (SC) (z (56) = 3.53, *p* = 0.004, *r* = 0.38). There was a clinically relevant shift for ANG which moved from the subclinical to nonclinical range as depicted in Fig. 2. Effect sizes for significant symptom reductions were in the small to medium range (0.2–0.5) for all domains with the largest effect on ANX (0.54).

**Table 2:** Percentage of children in the clinical range of the TSCC scales and subscales and change on trauma symptoms across T1 and T2 for Take Two clients between July 2007 and March 2019 (*n* = 91)

TSCC scales and subscales	T1		T2		Mean difference test^g^			Clinical movement
	Mean (SD)	% above clinical cut-off	Mean (SD)	% above clinical cut-off	t / z	*p*	Effect size	
Anxiety^e^	55.9 (11.2)	19.8	50.0 (10.2)	7.7	5.13^a^	< 0.001	0.54^c^	Subclinical-subclinical
Depression^e^	53.9 (12.7)	18.7	51.1 (10.3)	11.0	2.18^a^	.032	0.23^c^	Subclinical-subclinical
Anger^e^	53.7 (10.1)	14.3	49.7 (8.8)	4.4	3.84^b^	< 0.001	0.38^d^	Subclinical-nonclinical
Posttraumatic stress^e^	54.0 (9.5)	14.3	50.4 (9.6)	11.0	3.38^a^	.001	0.35^c^	Subclinical-subclinical
Dissociation^e^	55.8 (10.4)	26.4	52.6 (9.0)	9.9	3.08^a^	.003	0.32^c^	Subclinical-subclinical
Sexual concerns^f^	52.9 (13.6)	12.5	47.4 (11.5)	5.4	3.53^b^	.004	0.38^d^	Nonclinical-nonclinical

^a^t value

^b^z value

^c^effect size measured using Cohen’s *d*. Effect size estimates interpreted as small (*d* = 0.0–0.20), medium (*d* = 0.30–0.50), and large (*d* = 0.60–0.80)

^d^measured by $\mathrm r=\mathrm z/\surd n$ where 0.1 (small effect), 0.3 (moderate effect) and 0.5 and above (large effect)

^e^ 50–64: subclinical; > 64: clinical

^f^Sexual Concerns, Sexual Concerns-Preoccupation, Sexual Concerns-Distress: > 69 is clinical

^g^Cutoff for statistical significance was set at *p* < .008

Results from the McNemar test show that at T1, symptom scales above the clinical cut-off ranged from 12.5 to 26.4% and at T2 ranged from 4.4 to 11.0%, showing significant clinical symptom reduction in ANX (19.8% at T1 and 7.7% at T2), ANG (14.3% at T1 and 4.4% at T2), and DIS (26.4% at T1 and 9.9% at T2).

### TSCYC Cohort

**Fig. 3:**
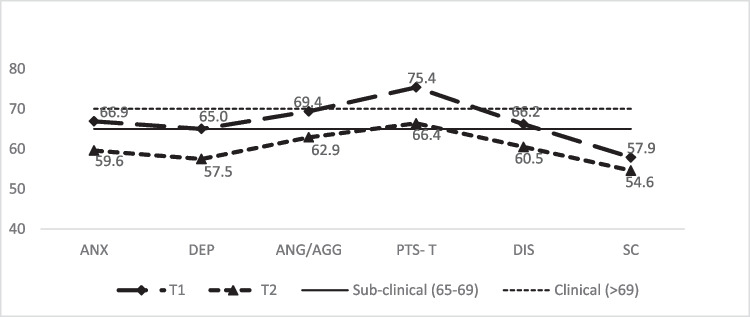
TSCYC Clinical change in symptoms from T1 to T2 in Take Two clients

In Table 3, paired sample *t*-test and Wilcoxon signed-rank test show a significant reduction in trauma symptoms from T1 to T2 in the following scales and subscales: ANX (*z* (144) = 5.48,* p* < 0.001, *r* = 0.46), DEP (*z* (144) = 5.35, *p* < 0.001, *r* = 0.45), anger/aggression (ANG/AGG) (*t* (144) = 5.64, *p* < 0.001, *d* = 0.47), DIS (*z* (144) = 4.09, *p* < 0.001, *r* = 0.34) and posttraumatic stress total (PTS-T) (*z* (144) = 6.36, *p* < 0.001, *r* = 0.53). There was a clinically relevant shift for ANG/AGG which moved from the clinical to nonclinical range as depicted in Fig. 3. PTS-T moved significantly from clinical to subclinical categories, while ANX, DEP and DIS moved from subclinical to nonclinical categories (Fig. 3). Effect sizes for significant symptom reductions were in the small to large range (0.20–0.53) on all domains with PTS-T the largest at 0.53.

**Table 3:** Percentage of children in the clinical range of the tscyc scales and subscales and change on trauma symptoms across T1 and T2 for Take Two clients between July 2007 and March 2019 (*n* = 144)

TSCYC scales and subscales	T1		T2		Mean difference test^f^				Clinical movement
	Mean (SD)	% above clinical cut-off^e^	Mean (SD)	% above clinical cut-off^e^	t/z		*p*	Effect size	
Anxiety	66.9 (16.5)	38.9	59.6 (15.2)	20.8	5.48^b^	< 0.001		0.46^d^	Subclinical-nonclinical
Depression	65.0 (16.2)	36.8	57.5 (14.2)	19.4	5.35^b^	< 0.001		0.45^d^	Subclinical-nonclinical
Anger/Aggression	69.4 (17.5)	46.5	62.9 (16.5)	34.0	5.64^a^	< 0.001		0.47^c^	Clinical- nonclinical
Posttraumatic stress—Total	75.4 (17.8)	58.3	66.4 (17.1)	31.9	6.36^b^	< 0.001		0.53^d^	Clinical-subclinical
Dissociation	66.2 (18.2)	34.7	60.5 (16.6)	24.3	4.09^b^	< 0.001		0.34^d^	Subclinical-nonclinical
Sexual concerns	57.9 (18.9)	17.0	54.6 (15.5)	9.2	2.33^b^	.020		0.20^d^	Nonclinical-nonclinical

^a^t value

^b^z value

^c^effect size measured using Cohen’s *d*. Effect size estimates interpreted as small (*d* = 0.0–0.20), medium (*d* = 0.30–0.50), and large (*d* = 0.60–0.80)

^d^measured by r = z/$\surd n$ where 0.1 (small effect), 0.3 (moderate effect) and 0.5 and above (large effect)

^e^ > 69: clinical

^f^Cutoff for statistical significance was set at *p* < .008

Results from the McNemar test reveal the highest clinical scores at T1 were in ANG/AGG (46.5%) and PTS-T (58.3%). At both T1 and T2, the most frequent clinically elevated scales were PTS-T (T1: 58.3%; T2: 31.9%) and ANG/AGG (T1: 46.5%; T2: 34.0%).

### TSCYC and Developmental Adversity (NMT™) Cohort

Results (Table 4) from the paired t-test shows a significant reduction on all trauma scales except for DIS and SC in children who experienced moderate to severe adversity as recorded through the NMT™ Part A during infancy. Significant improvements following treatment were found in ANX (*t* (102) = 4.87,* p* < 0.001, *d* = 0.48), DEP (*t* (102) = 3.71, *p* < 0.001, *d* = 0.37), ANG/AGG (*t* (102) = 4.56, *p* < 0.001, *d* = 0.45), PTS-T (*t* (102) = 5.91, *p* < 0.001, *d* = 0.59). Clinical to nonclinical movement occurred for ANG/AGG and clinical to sub-clinical movement occurred for PTS-T. On all domains, effect sizes for the significant symptom reduction ranged between small to medium (0.15–0.59). The McNemar test shows PTS-T (T1: 57.8%; T2: 32.4%) and ANG/AGG (T1: 48.0%; T2: 33.3%) were the most clinically elevated scale at both T1 and T2.

**Table 4:** Mean TSCYC scores of children in the clinical range across T1 and T2 with moderate to severe experiences of adversity in infancy according to NMT Metric Part A (*n* = 102)

Developmental adversity cohort	TSCYC scales and subscales	T1		T2		Mean difference test^c^			Clinical movement
		Mean (SD)	% above clinical cut-off^d^	Mean (SD)	% above clinical cut-off^d^	t	*p*-value	Effect size^a^	
Infancy total moderate/severe score	Anxiety	67.7 (16.6)	40.2	60.3 (15.5)	23.5	4.87	< 0.001	0.48	Subclinical-nonclinical
	Depression	64.6 (16.4)	35.3	57.9 (14.7)	20.6	3.71	< 0.001	0.37	Nonclinical-nonclinical
	Anger/Aggression	69.5 (17.1)	48.0	63.2 (16.8)	33.3	4.56	< 0.001	0.45	Clinical- nonclinical
	Posttraumatic stress—Total	75.7 (17.1)	57.8	66.8 (17.4)	32.4	5.91	< 0.001	0.59	Clinical-subclinical
	Dissociation	65.3 (18.3)	32.4	60.2 (16.7)	22.5	2.64	.010	0.26	Subclinical-nonclinical
	Sexual concerns	58.1 (19.7)	16.8	55.2 (17.0)	12.9	1.47	.145	0.15	Nonclinical-nonclinical

^a^Effect size measured using cohen’s *d*

^a^Effect size estimates interpreted as small (*d* = 0.0–0.20), medium (*d* = 0.30–0.50), and large (*d* = 0.60–0.80)

^c^Cutoff for statistical significance was set at *p* < .008

^d^ > 69: clinical

## Discussion

This study found that Take Two ITS interventions were associated with a significant reduction in client trauma symptoms. Outcomes were strongest in the younger TSCYC cohort, regardless of the severity of their adverse experiences in infancy. These results replicated the pattern of symptom improvement found in 2010 (Frederico et al., 2010) but with stronger results: significant reductions (*p* < 0.001) were reported across all TSCC and TSCYC scales in this study compared to significant reductions in some scales in 2010 (TSCC: anxiety, depression, anger (*p* < 0.01), posttraumatic stress (*p* < 0.05) and TSCYC: anger (*p* < 0.05)).

One notable difference in client demographics since 2010 is the increased number of children living in kinship care (Table 1). This is consistent with Child Protection statistics and policies (Australian Institute of Health & Welfare, 2023), which include a stronger emphasis on kinship care, especially for Aboriginal and Torres Strait Islander children, alongside increased demand for out-of-home-care placements and a shortage of foster care placements (Boetto, 2010; Smart et al., 2022). Kinship care may improve the stability of placements for older children (Farrugia & Joss, 2021) and has been associated with reduced behavioural problems in children aged six to 17.5 years (Wu et al., 2015), especially when supports such as mental health care are available (Sakai et al., 2011). The higher number of children in kinship care in this study may influence the stronger effect sizes reported, with the impact of placement type warranting further research.

While significant improvement in TSCC trauma symptoms was found across every clinical scale, anxiety showed the largest effect size (*d* = 0.54), consistent with similar studies (Dauber *d* = 0.6, Ford* d* = 0.61) (Dauber et al., 2015; Ford et al., 2012). A study on ARC (*n* = 481) utilising the TSCC to assess 6–12 year old children in a multi-site adoption program found significant reductions in anxiety, which continued declining 12 months post treatment (Hodgdon et al., 2016). The authors noted the importance of a stable, supportive carer in complex trauma treatment, a factor present for 60.1% of participants in adoptive care. In this study only 1.1% (TSCC) and 0% (TSCYC) of the cohort were in permanent care, as children on existing Permanent Care Orders are not eligible for ITS. Despite these differences, the similar focus on self-regulation skills and positive attachments may have contributed to reduced anxiety, as reported previously (Cox et al., 2021b).

Significant trauma symptom reduction following treatment for developmental trauma is reported in similar TSCC studies with doses of approximately 6–12 months (Bartlett et al., 2018; Dauber et al., 2015). Dauber (2015) found that posttraumatic stress symptoms improved in accordance with length of treatment. While this study did not measure treatment length, we found positive changes after a maximum 16-month duration. There is no consensus on effective treatment length, however children exposed to developmental trauma such as abuse and neglect likely require longer than standard interventions (Cohen et al., 2012).

This study revealed a contrast between TSCC and TSCYC results when comparing Mean T-scores, with younger children (TSCYC) showing higher T1 scores across all scales and a greater reduction in clinically significant symptoms. This was also observed in 2010, reflecting increased benefits of Take Two ITS interventions for younger children and/or potential discrepancies between self and carer-reports. Report differences are noted by others with approximately 20% of at-risk youth under-responding on the TSCC (Butcher et al., 2014), indicating that self-reported trauma symptomatology in the TSCC may be underestimated.

For the TSCYC cohort, the most significant reduction in symptoms (from clinical to nonclinical) was seen in Anger/Aggression (Fig. 3). Anger/Aggression also showed clinically meaningful change (sub-clinical to non-clinical) in the TSCC cohort (Fig. 2), and a similar result was found in 2010. Anger and maladaptive behaviour are common reasons for referral to child and adolescent mental health services (Sukhodolsky et al., 2016). For child protection clients, anger is often a response to victimization, adults failing to protect them, a sense of parental abandonment and the self-regulation difficulties that arise from developmental trauma (Cohen et al., 2010; Lavi et al., 2019). Trauma focused therapies that begin by establishing safety and addressing emotion-regulation skills (Cook et al., 2005; Thornback & Muller, 2015) are likely to have a large effect on both anger and anxiety as demonstrated in this study. Empathic attunement may decrease hyper-arousal and increase safety contributing to a reduction in anger and anxiety (Paivio & Laurent, 2001). The positive experience and impacts of kinship care, especially when this care is supported to be relationally rich (Hambrick et al., 2019a), might also contribute to these results.

The largest effect in the younger TSCYC cohort was for PTS-T (*d* = 0.53), supporting the hypothesis that Take Two ITS contributes to a reduction of trauma symptoms in children even when their adverse experiences in infancy have been severe. These results suggest this cohort is reaching the end phase of treatment, where trauma processing and integration occurs (Ford, 2021). Further research is needed to understand the mechanisms of this change, with controls for treatment specifics and placement stability.

Therapeutic psychoeducation provided to carers may also account for this study’s positive findings, with carers’ increased understanding of trauma-related behaviours leading to positive changes in children’s presentation and relational health (Cox et al., 2021b). Young children may present with a lower trauma load due to a reduced cumulative effect of adverse events; they often have lower attrition rates than older clients (Wamser-Nanney & Steinzor, 2016); and they were more likely to be living in kinship care in this study. All these factors may assist their responsiveness to treatment.

Even when children experienced moderate to severe adversity in infancy, Take Two interventions were associated with a statistically and clinically significant reduction in anxiety, depression, anger, and posttraumatic stress symptoms (Table 4). These results present a strong argument for the benefits of interventions for children earlier in life as the positive treatment outcomes found in this study likely reduce the severity and chronicity of problems seen in school-age children and adolescents with complex trauma (Cook et al., 2005). Longitudinal follow up of this cohort would add insight into the long-term experience, impact of adversity and value of interventions for young children.

## Strengths and Limitations

The longitudinal nature of this data collection indicates a pattern of improved outcomes over time as the Take Two program has evolved. A large sample size allowed for more rigorous inclusion criteria; however, several limitations hampered external validity, and the findings must be considered cautiously. The lack of a control group in this study renders the findings descriptive as they may not reflect true treatment effects. The lack of treatment specifics also restricts our understanding of which interventions contributed to positive effects. The individualised treatment plans implemented at Take Two and the retrospective nature of this study, meant that treatment length varied. Future studies would benefit from prospective comparison designs that identify a consistent dose, duration, and treatment type while addressing the ethical challenges of restricting treatment for developmental trauma.

The study’s use of the NMT™ Part A metric to obtain developmental adversity data for the cohort controlled for severity but not for adversity type which would enhance future studies. A small sample in the TSCC Developmental Adversity cohort prevented the inclusion of this data, restricting results to the TSCYC cohort.

One of many challenges in working with children involved in the Child Protection system has been ongoing provision of effective treatment to children who lack consistent caregivers (Frederico et al., 2019). Placement instability was not captured in this study and is likely to be a common impediment to successful outcomes for children. Future research on outcomes for children in out-of-home-care could consider placement stability alongside placement type and adversity history as these factors can impede treatment success (Rock et al., 2015).

## Implications for Clinical Practice

Implications for clinical practice are made cautiously due to the limitations of this study. The findings suggest that individualised treatments, informed by comprehensive assessments, may improve outcomes for children with developmental trauma symptoms. The use of multiple therapies at Take Two enables interventions to be matched to client symptoms in a precision approach increasingly endorsed in trauma treatment (Dauber et al., 2015; Ford, 2021). Intervening earlier in life further improved outcomes in this study, indicating the importance of encouraging stakeholders (Child Protection in this study) to refer infants. Kinship care may also have been beneficial in achieving positive outcomes for young children: supporting kinship relationships with therapies such as CPP (Lieberman et al., 2011) is recommended to provide the relationally rich environment that facilitates children’s recovery (Hambrick et al., 2019a).

## Contribution to Research and Recommendations

This study contributed to emerging research on developmental trauma treatment in usual care settings (Bartlett et al., 2018; Dauber et al., 2015), while acknowledging the inherent challenges of demonstrating effectiveness for developmental trauma interventions (Ford, 2021). The term developmental trauma, used in this paper to encompass the experience and impact of multiple types of trauma, aptly describes the phenomenon observed over the years in our clinical sample. The NMT™ is one of few measures that considers children’s age at time of adversity; the use of the NMT™ metric Part A to link adversity experiences to trauma outcomes here provides an innovative addition to developmental trauma research. Future research could build upon this investigation with increased samples enabling inclusion of information regarding adversity type, intervention, and the impact of relationships on therapeutic outcomes.

A lack of assessment tools specific to developmental trauma has led to the use of multiple clinical measures at Take Two. Future studies could examine trauma symptoms alongside the impact on children’s development and adaptive behaviour. Given the paucity of research on developmental trauma treatment in community settings, more studies such as this are recommended.

## Conclusion

The work of Take Two ITS appears to have contributed to significantly reduced trauma symptoms, especially for younger children, including when adverse experiences during their infancy were moderate to severe. Results for younger children suggest that therapeutic interventions early in life may lead to the greatest reductions in reported symptoms. While anger and posttraumatic stress were the most reduced symptoms in the younger age-group, anxiety appears to be greatly influenced by trauma-specific treatment in the older age-group, a finding reported elsewhere (Dauber et al., 2015; Ford et al., 2012). Overall, these findings point to the benefits of NMT™ informed, individually tailored interventions delivered early in life, while contributing to the developing body of knowledge on effective developmental trauma interventions.

## Data Availability

Not applicable.
